# Solution for the hot spots in adaptive radiotherapy planning for cervical cancer: a case report and review of literature

**DOI:** 10.3389/fonc.2026.1862463

**Published:** 2026-06-26

**Authors:** Peng Jiang, Xiang Wang, Shubao Cui, Chengyu Ren, Zilu Wang, Yongchun Zhang, Xiaoqian Wu

**Affiliations:** Department of Radiation Oncology, The Affiliated Hospital of Qingdao University, Qingdao, China

**Keywords:** cervical cancer, Ethos, hot spots, online adaptive radiotherapy, treatment planning

## Abstract

The Ethos system (version 2.0, Varian Medical Systems, Palo Alto, CA) is an online adaptive radiotherapy (ART) platform that can automatically replan based on the daily anatomical structure and achieve precise dose delivery. However, the highly automated online ART workflow may sometimes lead to excessive dose accumulation within the target in adaptive plan. This article reports a cervical cancer case in which hot spots appeared in the adaptive plan during adaptive treatment session. In these adaptive plans with hot spots, the edge of target was close to the multi-leaf collimator (MLC) boundary, resulting in limited leaf movement and reduced modulation flexibility. Therefore, we propose an improved online ART workflow to correct the deviation of the target, which effectively reduced the risk of hot spots in adaptive plan. This article discusses the problem occurrence, clinical response strategies and improved ART workflow, aiming to provide reference for improving the quality of adaptive plan and safety and stability of ART.

## Introduction

Cervical cancer is a common malignant tumor among women, and radiotherapy is a crucial treatment method (1). However, accuracy dose delivery remains challenging because of the significant inter-fractional anatomical changes in the target and surrounding organs at risk (OARs) (1, 2). The tumor regression, changes in bladder and rectal filling, and bowel motion can alter the position of target (3). These anatomical changes may lead to overestimation of target coverage and underestimation of OARs dose. Although conventional image-guided radiotherapy (IGRT) improves setup accuracy, it is generally unable to fully compensate for these daily anatomical changes.

Online adaptive radiotherapy (ART) offers a solution by reoptimizing treatment plan based on daily anatomical images (4, 5). The Ethos system (version 2.0, Varian Medical Systems, Palo Alto, CA) combines high-quality cone-beam computed tomography (CBCT) imaging system with an automated online adaptive workflow. For each fraction, it generates a scheduled plan recalculated on the current session image and anatomy, and a fully reoptimized adaptive plan (6). This approach improves target coverage and protects OARs when significant anatomical changes occur, while maintaining clinical workflow efficiency (7).

Nevertheless, the highly automated planning may introduce dosimetric uncertainties. The adaptive plan quality can be influenced by auto-segmentation error, and suboptimal optimization objectives and image artifacts (8–11). These factors may interfere with the dose calculation and result in insufficient target coverage. In our clinical experience, we found an unexpected hot spot in the adaptive plan of a cervical cancer patient treated with the Ethos system, raising concern regarding plan robustness and treatment safety.

To our knowledge, this is the first report of hot spots formation in the Ethos adaptive plan for cervical cancer. In this study, we investigate the mechanism underlying the hot spots and propose an improved online ART workflow to mitigate hot spots occurrence in the adaptive plan. This work may help improve the robustness, dosimetric quality and clinical safety of online ART for cervical cancer.

## Case presentation

### Clinical presentation

The patient was a 58-year-old female who presented with irregular vaginal bleeding in Dectmber 2025. The gynecological examination revealed a large amount of vaginal discharge. The cervix surface was smooth but enlarged and firm, measuring approximately 6 cm × 7 cm. The bilateral parametria were elastic. The ultrasound examination revealed an enlarged cervix and a hypoechoic mass with detectable blood flow. Pelvic magnetic resonance imaging (MRI) showed an enlarged uterus, and a heterogeneous mass in the anterior cervix measuring approximately 51mm × 65 mm× 58 mm. The lesion demonstrated heterogeneous hyperintensity on diffusion-weighted imaging (DWI) and extended superiorly into the uterine cavity and inferiorly toward the upper one-third of the vagina. No obvious abnormal signal was observed in the bilateral adnexal. The colposcopy-guided cervical biopsy confirmed poorly differentiated squamous cell carcinoma. Immunohistochemistry results showed that: P16(+), Ki-67(+), Estrogen Receptor (ER) (-), Progesterone Receptor (PR) (+), Vimentin (-), P53(+, mutant type), NapsinA (-). Positron emission tomography-computed tomography (PET/CT) showed increased uptake in the cervical lesion (SUV_max_: 7.1) and mildly elevated metabolism in the left common iliac lymph nodes (SUV_max_: 2.1), indicating nodal involvement. According to the International Federation of Gynecology and Obstetrics (FIGO), the tumor was staged as IIIC1.

This patient initially received one cycle of chemotherapy combined with immunotherapy before radiotherapy. During external beam radiotherapy, the patient subsequently received four additional cycles of weekly cisplatin at a dose of 40 mg/m². Immunotherapy consisted of cadonilimab at a dose of 500 mg every 3 weeks for three cycles. External beam radiotherapy was delivered using Ethos with ART followed by high-dose-rate intracavitary brachytherapy (HDR-ICBT). 24Gy in 4 fractions once a week was delivered to point A in HDR-ICBT.

### Simulation

Approximately 1.5 hours before the computed tomography (CT) simulation, the patient emptied the bladder and rectum, and then drunk approximately 500mL of water to achieve a bladder volume of 300-500mL. This patient was positioned in a foot-first prone and immobilized using a thermoplastic mask.

Contrast-enhanced CT simulation was performed using a 16-slice Philips Brilliance Big Bore CT simulator with a slice thickness of 5 mm. Iodixanol was administered intravenously at a volume of 70 mL and an injection rate of 2 mL/s. Subsequently, the acquired planning CT images were imported into Eclipse treatment planning system (version 15.1, Varian Medical Systems, Palo Alto, CA, USA) for delineation.

### Delineation and treatment planning

Based on the clinical presentation and planning CT images, the clinical target volume (CTV) included the cervix, uterus, upper vagina, parametria, and regional lymph nodes (common, internal and external iliac, obturator, and presacral). A uniform 2 mm expansion was applied to the CTV to generate the planning target volume (PTV). The primary gross target volume (PGTV) was defined as the involved lymph node adjacent to the left common iliac artery.

The prescribed dose was 47.6 Gy in 28 fractions for PTV, with simultaneous integrated boost of 60.2 Gy delivered to PGTV. The external body contour and OARs were automatically generated by Ethos treatment planning system. The dose constraints for OARs were defined as follows: spinal cord: D_max_ ≤ 40 Gy, cauda equina: D_max_ ≤ 50Gy, small intestine: D_max_ ≤ 52Gy, bladder: V_50Gy_ ≤ 10%, rectum: V_50Gy_ ≤ 10%, and left and right femoral heads: D_max_ ≤ 45Gy. D_max_ was defined as the point maximum dose within the target or OARs.

The volumetric modulated arc therapy (VMAT) plan using 6 MV flattening-filter-free (FFF) photons was designed by Ethos treatment planning system. The clinical goals required that at least 95% of the target be covered by the prescribed dose, and that dose constraint for OARs be met. Dose distribution was optimized by adjusting clinical goal priorities in the dose preview and plan was automatically generated in the plan review. The final plan was clinically and technically approved by the radiation oncologist and physicist, and used as a reference plan.

### Ethos adaptive treatment session workflow

**Figure 1:**
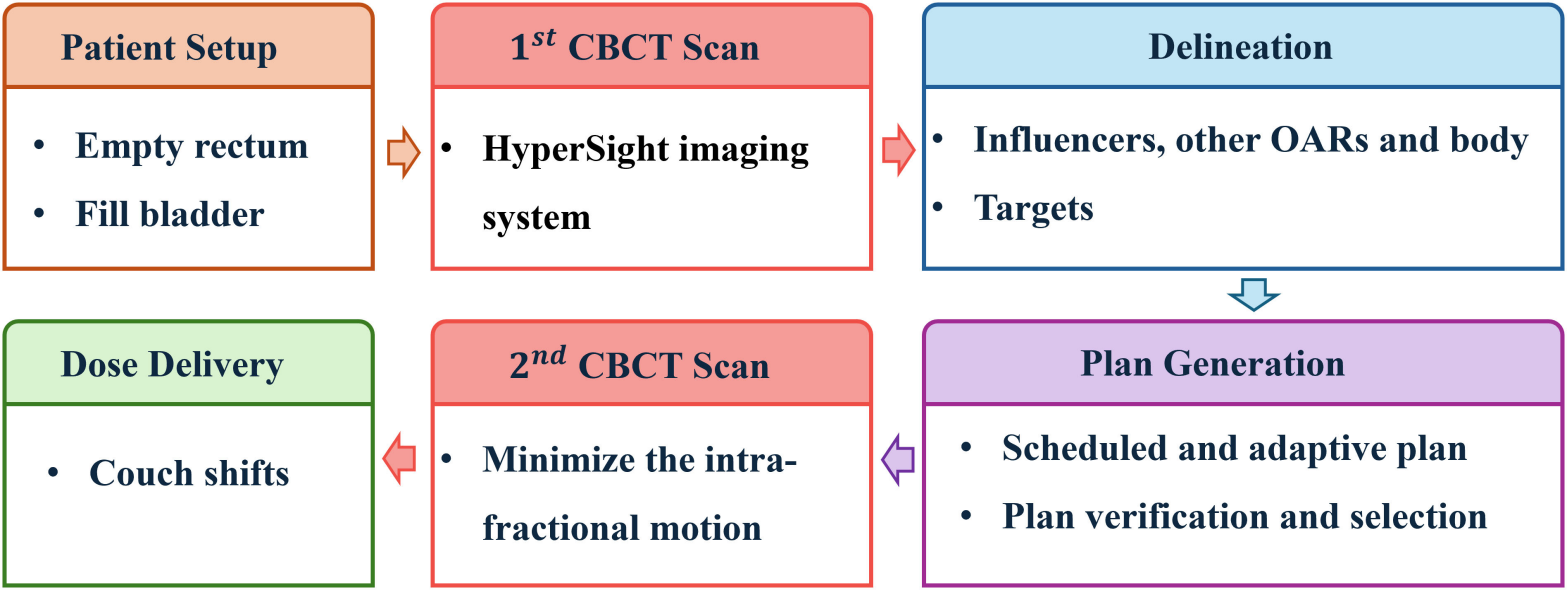
The workflow of adaptive treatment session using Ethos.

The Ethos ART workflow was shown in **Figure 1**. Prior to each ART session, the patient emptied the rectum and filled the bladder as during CT simulation. After initial setup, CBCT images were acquired using HyperSight imaging system and accepted by radiation oncologist. Influencer structures, other organs and external body contour were automatically generated by Ethos system and the radiation oncologist reviewed and edited them if necessary. All targets were deformed and registered from planning CT to CBCT images. The radiation oncologist also needed to review all targets and edit them if necessary. The scheduled plan and adaptive plan were automatically generated and online dose calculation verification for both plans were conducted using Mobius 3D system. After plan evaluation, the optimal plan was selected and approved for treatment of the current session. To minimize the intra-fractional motion, a second CBCT scan was performed and couch shifts were applied before dose delivery.

### Hot spots occurrence during adaptive planning

**Figure 2:**
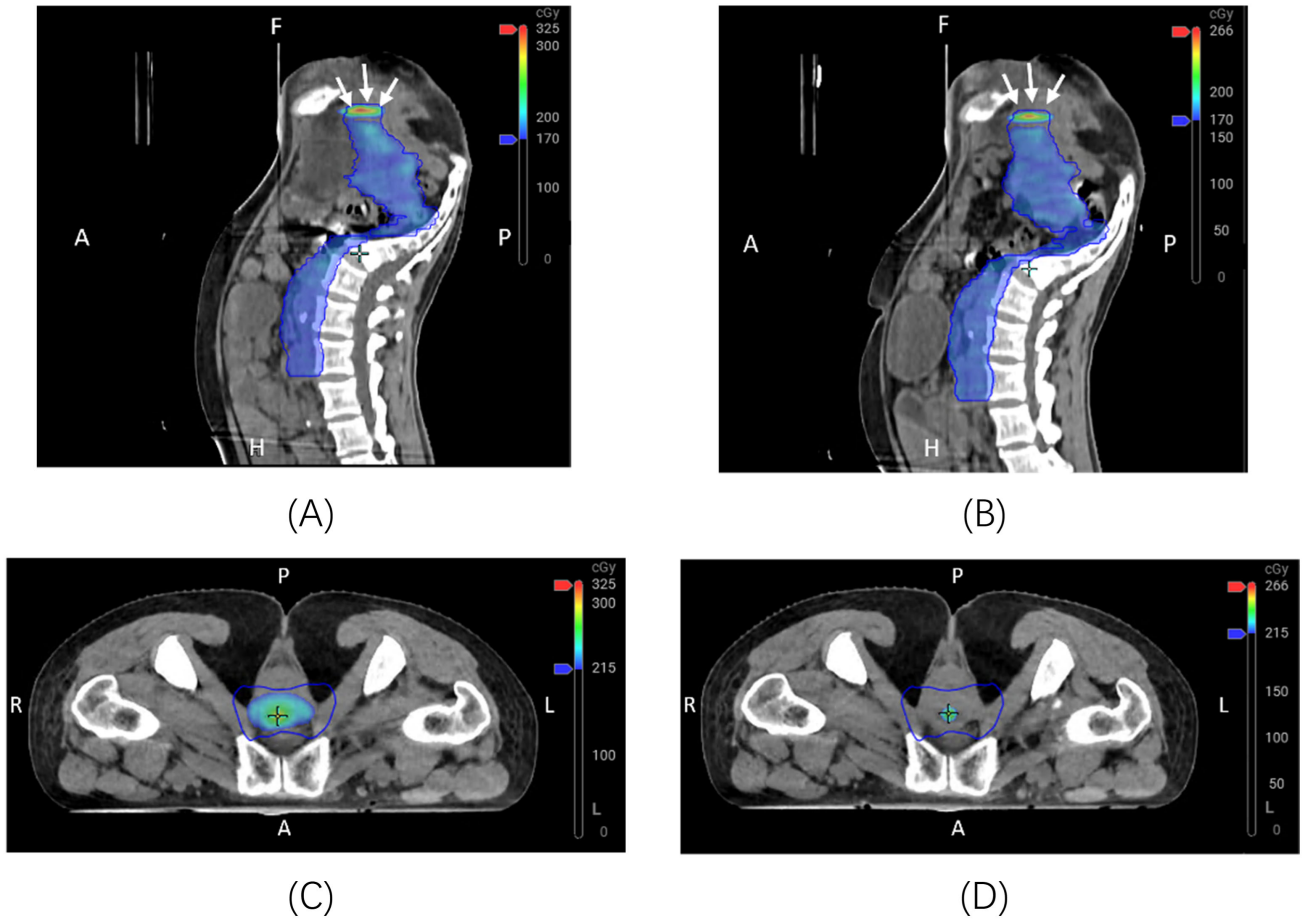
Hot spots observed in the second **(A, C)** and forth **(B, D)** adaptive plans.

During the second adaptive treatment session, hot spots were observed in the adaptive plan with the PTV D_max_ of 325 cGy, corresponding to 142% of the prescribed dose. The hot spots recurred during the fourth adaptive treatment session, in which the PTV D_max_ of 236 cGy. In both sessions, the hot spots were located at the superior edge of PTV in the sagittal plane, as shown in **Figures 2A, B**. The cross-sectional dose distributions for the 215 cGy isodose within PTV are shown in **Figures 2C, D**.

Compared with the adaptive plans, the scheduled plans demonstrated improved dose homogeneity, as shown in **Supplementary Figure 1**. Quantitative dosimetric comparisons of targets between the scheduled and adaptive plans are summarized in **Table 1**. As illustrated in **Supplementary Figure 2**, the adaptive plans had unacceptable high-dose spots within the PTV, whereas target coverage was comparable between the two plans. Accordingly, the scheduled plans were selected for treatment delivery in the second and fourth fractions.

**Table 1:** Dosimetric comparison of targets between scheduled plans and adaptive plans in the second and fourth adaptive treatment sessions.

Session	Plan type	PTV D_95%_ (%)	PGTV D_95%_ (%)	PTV D_max_ (cGy)	PGTV D_max_ (cGy)
2	Scheduled	99.7	98.7	237	237
	Adaptive	98.7	101.1	325	236
4	Scheduled	99.3	101.1	236	236
	Adaptive	100.6	100.7	266	235

By contrast, during the first and third adaptive treatment sessions, no hot spots were observed in the adaptive plans. In these sessions, the adaptive plans showed better conformity and dose homogeneity than the scheduled plans and were selected for treatment.

### Cause analysis

To investigate the cause of the hot spots observed in the adaptive plans, several potential factors were evaluated.

Firstly, contour-related factors were excluded. Influencer structures including the bladder, rectum and uterus, and other organs were carefully reviewed. The PTV and PGTV were also reviewed and edited by radiation oncologists during the adaptive treatment sessions. All derived structures were generated according to the predefined derivation rules and no non-derived structures were used for plan optimization.

Secondly, the impact of clinical goals was evaluated. After the second adaptive treatment session, a new revision of the RT intent with modified clinical goals was generated. In the revised plan, OAR constraints were relaxed, while stricter constraints were applied to derived structures to improve dose distribution. The comparison between the original and modified clinical goals is shown in **Table 2**. In addition, to improve modulation capability, the VMAT field arrangement was changed from two full arcs to three full arcs. Despite these modifications, hot spots still recurred in the fourth adaptive treatment session. This suggested that hot spot formation was not primarily attributable to the clinical goals and field arrangement.

**Table 2:** The comparison of original and modified clinical goals.

Category	Structure	Original clinical goals	Priority	Modified clinical goals	Priority
Organs	Femur head & neck left	D_0.03cm3 ≤_ 3800 cGy (*Var* D_0.03cm3 ≤_ 4200 cGy)	3	D_0.03cm3 ≤_ 4000 cGy (*Var* D_0.03cm3 ≤_ 4400 cGy)	3
	Femur head & neck right	D_0.03cm3 ≤_ 3800 cGy (*Var* D_0.03cm3 ≤_ 4200 cGy)	3	D_0.03cm3 ≤_ 4000 cGy (*Var* D_0.03cm3 ≤_ 4400 cGy)	3
The derived structures	PTV-(PGTV + 1.5cm)	D_0.03cm3 ≤_ 5050cGy (*Var* D_0.03cm3 ≤_ 5188 cGy)	1		
	PTV-(PGTV + 2.5cm)			D_0.03cm3 ≤_ 5093 cGy (*Var* D_0.03cm^3^_ ≤ 5188 cGy) D_max_ ≤ 5113 cGy (*Var* D_max_ ≤ 5208 cGy)	1

**Figure 3:**
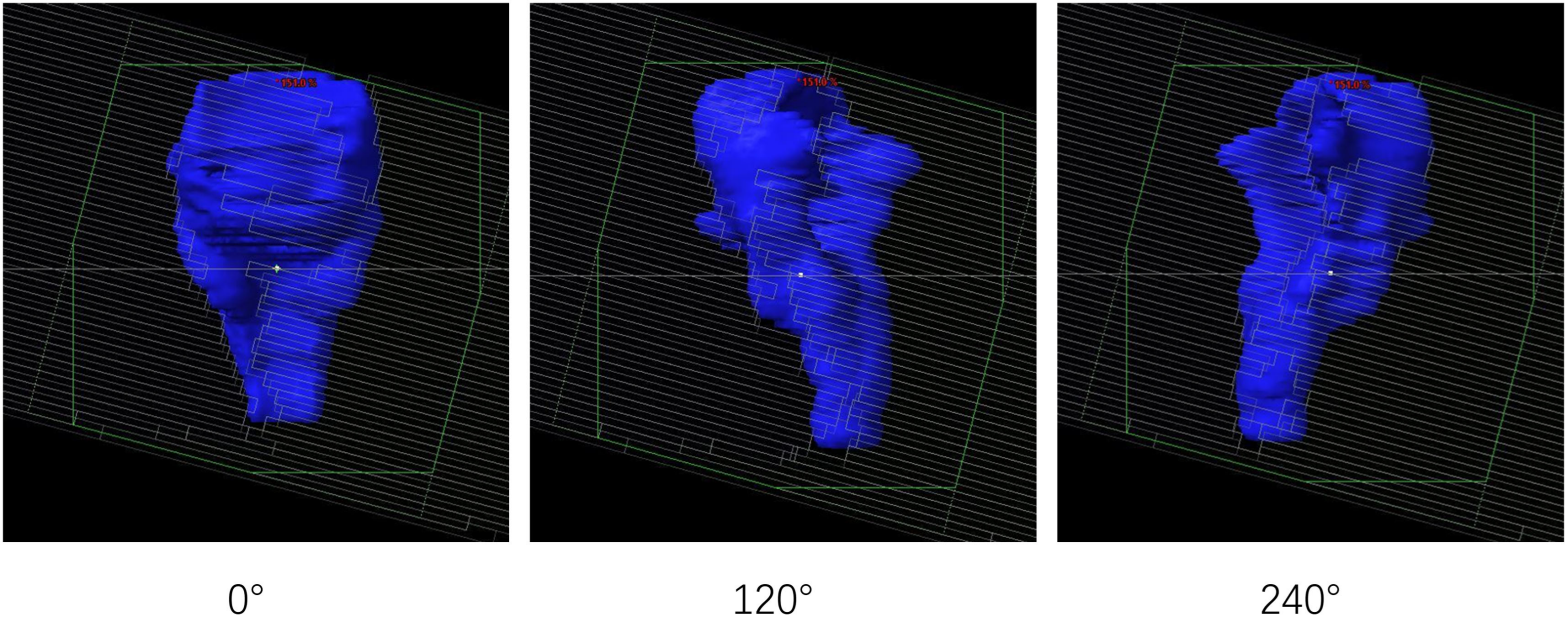
The geometric relationship of the multi-leaf collimator (MLC) and PTV at different gantry angles in beam eye view (BEV).

Lastly, the geometric relationship between the target and the radiation field was analyzed. In the reference plan, the treatment isocenter was placed at the geometric center of the PTV. In the adaptive plans, the treatment isocenter at (0, 0, 0) corresponds to the imaging isocenter. In the scheduled plans, couch shifts are calculated to align the daily target with that of the reference plan. In this case, the PTV length was 24 cm. The treatment isocenter located 12 cm from both the superior and inferior edge of the PTV in the reference plan. In the second and fourth scheduled plans, the treatment isocenter shifts were −1.61 cm and −1.57 cm respectively. In the adaptive plans with hot spots, the treatment isocenter was approximately 13 cm from the superior edge of PTV and 11 cm from the inferior edge, indicating the treatment isocenter significantly deviated from the intended target isocenter. The geometric relationship between the MLC and PTV at different gantry angles in beam eye view (BEV) is shown in **Figure 3**.

When sufficient leaf travel margin was available on both sides of the target, the optimizer had adequate modulation flexibility to achieve target coverage and dose homogeneity. However, after target displacement during adaptive treatment, one side of the PTV approached the MLC boundary. The corresponding leaf group reached its maximum travel limit. This mechanical constraint reduces the optimizer’s ability to create dose fall-off outside the target. To maintain target coverage, the optimizer compensated by increasing beam intensity, resulting in excessive dose deposition within the PTV and hot spot formation.

In conclusion, these findings indicate that hot spots were primarily caused by reduced modulation flexibility due to mechanical constraint. This case highlights the importance of the geometric relationship between the target and MLC to avoid unexpected hot spots, and improve the robustness of the plan in online ART workflow.

### ART workflow optimization

During the sixth adaptive treatment session, hot spots reappeared in the adaptive plan. To investigate whether the hot spots were related to the deviation of the center of target from the treatment isocenter, this adaptive treatment session was closed and a CBCT scan was acquired. After image registration of CBCT to the planning CT, radiation therapists corrected the patient setup to correct the deviation of target. The patient was shifted 1.5 cm in the superior direction, and the adaptive treatment session was restarted. Following repositioning, a new adaptive plan was generated, in which no hot spots were observed.

**Figure 4:**
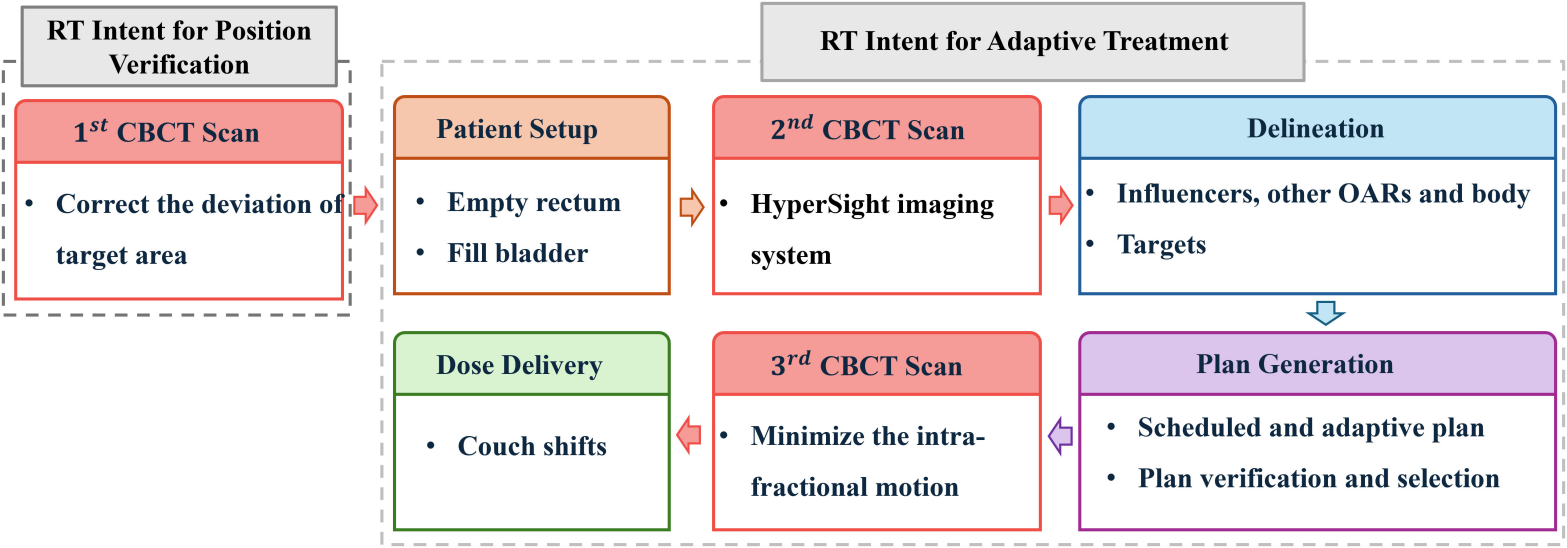
The improved adaptive treatment workflow.

To reduce the risk of hot spots during adaptive treatment session, an improved ART workflow was implemented, as shown in **Figure 4**. Two RT intents were prepared before the adaptive treatment session: one for position verification, and the other for adaptive treatment. First, a CBCT scan was acquired using the RT Intent for position verification. The CBCT images were registered with the planning CT to evaluate the positional difference along the Y-axis. The RT intent for adaptive treatment was then opened. The patient position was further corrected by applying the positional difference along the Y-axis before ART workflow proceeded.

These finding supports that the geometric relationship between the target and the MLC is a critical factor in hot spots formation. By maintaining the target isocenter close to the treatment isocenter, the optimized ART workflow reduced the risk of abnormal dose accumulation in the adaptive plan.

### Discussion and review of the literature

To our knowledge, this is the first reported case of hot spots appeared in an Ethos adaptive plan during adaptive treatment session caused by the edge of target approaching the MLC boundary. The reduced modulation flexibility leads the optimizer to increase beam intensity to maintain target coverage, resulting in excessive dose accumulation within the target. In our case, an improved ART workflow incorporating an additional CBCT scan and setup correction was proposed, effectively minimizing the deviation of the target and reducing the risk of hot spots in the adaptive plan. This observation suggests that the mechanical limits of beam shaping and delivery can affect the dosimetric quality of Ethos adaptive plan.

This finding has clinical significance because prior studies have shown that compared with scheduled plans, the adaptive plans generally have superior dosimetric performance with the use of Ethos (5, 7, 11). In the first prospective head-and-neck (H&N) experience, adapted plans improved PTV coverage and reduced mean hot spots relative to scheduled plan (8). Recent studies also have reported the clinical and dosimetric value of ART in reducing PTV margins, improving target coverage, sparing OARs and facilitating dose escalation and hypofractionation, especially in the treatment of cervical, bladder, and lung cancers (12–14). However, our case demonstrates that adaptive plan cannot completely eliminate the possibility of hot spots within the target. ART reduces anatomical differences between simulation and treatment, but still has problems such as incorrect delineation, optimization error and intra-fractional movement. Therefore, average dosimetric improvement does not mean that hot spots will not form during adaptive treatment session.

The Ethos optimization is driven by a prioritized list of clinical goals, and the adapted plan remains reoptimized by these clinical goals (6). Consequently, the reference plan quality and clinical directive template are important for plan quality. If the homogeneity control or hot spot suppression are insufficient in the plan directive, there may still be dose inhomogeneity or hot spots within the target in the adaptive plan. Rayn K et al. used dosimetric scorecard tool to tune Ethos directive templates, improving plan quality and efficiency in cervical cancer (9). For ART, a well-designed directive template is a key factor in ensuring the convenience and robustness of replanning. The stricter upper-dose constraints, and more explicit homogeneity goals should be applied if necessary.

Regarding the impact of different field arrangements on the plan quality, El-Qmache A et al. observed that Ethos IMRT plans provided better target coverage and conformity than VMAT for H&N patients, while VMAT plans showed inferior low-dose PTV coverage (15). They also found that the higher MU of Ethos IMRT plans have greater delivery complexity and require additional quality assurance attention.

The Ethos also utilizes artificial intelligence (AI) technology for auto-contouring, dose calculation and auto-planning. Although the ART workflow of Ethos is efficient with the assistance of AI, it introduces some “black-box” steps that still require manual review. For the H&N patients, inaccurate body contour needed attention because it affected both PTV cropping and dose calculation (8). For pelvic and abdominal tumors, inter-fractional bladder and rectal filling, uterus motion, and bowel gas vary substantially (10, 16), which makes the accuracy of auto-contouring particularly important. Zhang et al. reported the auto-contouring was generally clinically acceptable for cervical cancer, but the uterus, upper vaginal cuff, and lower nodal CTV are more likely to require careful review and correction (17).

Because patient anatomy may change during treatment, workflow efficiency is important when evaluating the dosimetric benefit of an adaptive plan. Previous studies reported Ethos ART procedural time was related to disease site, generally requiring about 15–35 minutes for the adaptive treatment session (18–21). In cervical cancer, if the treatment duration is too long, significant volume change in bladder and rectum may occur. The anatomy used for optimization may not match the anatomy at dose delivery. Accordingly, strict bladder and rectal preparation, efficient ART workflow, and position verification before beam-on are crucial measures, which affect the accuracy of the adapted dose distribution and dose delivery.

Although the quality of adaptive plan using Ethos is commonly discussed in terms of auto-contouring and re-optimization, the final dose distribution is also constrained by the delivery characteristics factors of the Ethos. Studies of the dual-layer MLC have shown low leaf transmission and measurable leaf-end effect, leaf-edge effect, and tongue-and-groove effects (22, 23). In addition, the dual-layer design introduces leaf-tip transmission and leaf-trailing effects, both of which can influence calculated and delivered dose near field edges (24). Overall, when the edge of target approaches the MLC boundary, the optimizer will be constrained by both algorithm and mechanical limitations, and try to meet the target coverage with finite leaf width, rounded leaf ends, edge effects, and reduced modulation flexibility. Even if the contours have been manually reviewed and the clinical goals are strict, there may still be hot spots in the adaptive plan due to mechanical limitations.

In radiotherapy plan, it is common to observe cranial and caudal edge enhancement, where the dose is slightly higher at the ends of the target volume as a result of a combination of physical and planning-related factors. These include optimizer attempts to maintain adequate target coverage, beam geometry end effects along the superior-inferior axis, and MLC-related effects near field boundaries. Therefore, these effects should be recognized as inherent to the planning and delivery process and should not automatically be regarded as planning errors or machine failures. Accordingly, the hot spots observed in this case may be interpreted as a multifactorial dose distribution phenomenon. Although the spatial relationship between the displaced target and the MLC boundary suggested a potential contribution from reduced modulation flexibility, other factors may also have contributed, including optimizer behavior, beam geometry end effects, MLC characteristics, and inter-fractional anatomical changes. To reduce this risk, adaptive plan optimization and review should include standard target coverage and organ-at-risk constraints, stricter D_2%_ or D_max_ objectives, increased homogeneity priority, and auxiliary hot spot-control structures at the edges of the target when appropriate. Our proposed workflow with an additional CBCT and setup correction avoids the edge of target close to the MLC boundary. In our clinical practice, if the patient’s positioning deviation is significant, then position verification is necessary before adaptive treatment session.

Finally, successful ART implementation in cervical cancer requires clear quality assurance measures (25). For large gynecological targets, initial plan design should consider the assessment of PTV-to-MLC boundary distance, maximum leaf travel, expected inter-fractional displacement, dose homogeneity, and anatomical changes. For the quality of adaptive plan, especially hot spots management, a practical approach is to combine robust planning templates, contour review, dose validation, and additional position validation. Under these conditions, ART can achieve personalized daily replanning while minimizing the risk of hot spots in adaptive optimization.

## Conclusion

In this report, we described a cervical cancer case in which hot spots appeared in Ethos adaptive plans during the adaptive treatment session. When the target approached the MLC boundary, limited leaf motion and reduced modulation flexibility contributed to excessive dose accumulation. An improved workflow with additional CBCT verification and setup correction helped reduce this risk. These findings indicate that the quality of both adaptive and conventional treatment plans may be influenced not only by standard plan objectives, but also by underlying mechanical constraints and the characteristics of ART workflow implementation, particularly in cases involving very long or ultra-long PTVs.

## Data Availability

The raw data supporting the conclusions of this article will be made available by the authors, without undue reservation.
