# Supplementary material for: Solution for the hot spots in adaptive radiotherapy planning for cervical cancer: a case report and review of literature
Source: Front Oncol. 2026 Jun 26;16:1862463. doi: 10.3389/fonc.2026.1862463 (PMC13351529; doi:10.3389/fonc.2026.1862463)
Supplement: Supplementary file 1 [file DataSheet1.docx]

***Supplementary Material***

1. **Supplementary Figures and Tables**
2. **Supplementary Figures**

| 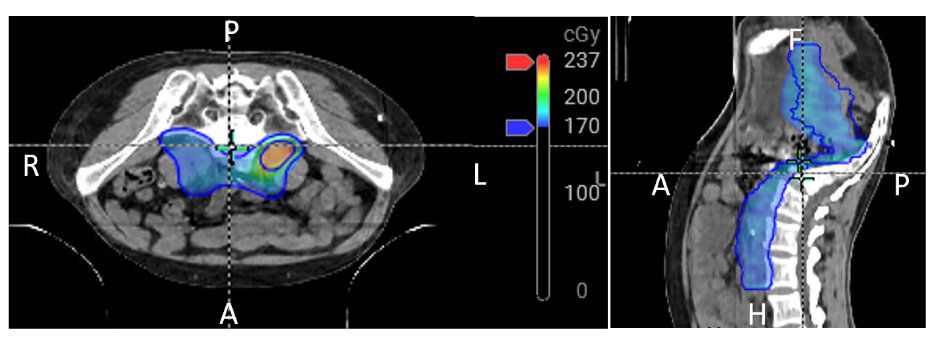 |
| --- |
| （A） |
| 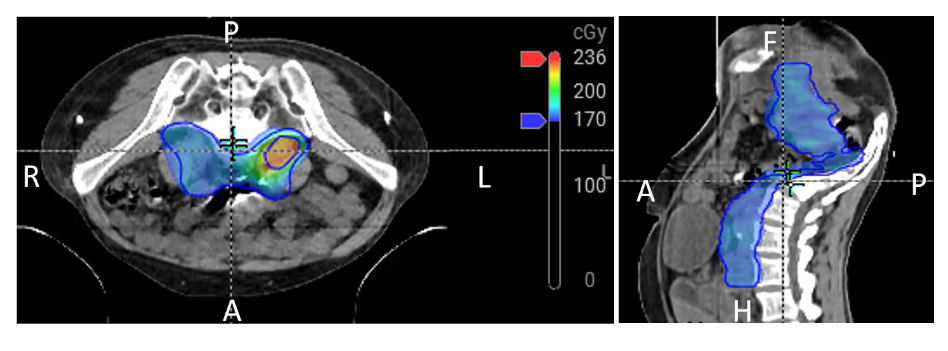 |
| （B） |

Supplementary Figure 1. Dose distribution of scheduled plans in the second (A) and fourth (B) adaptive treatment sessions.

| 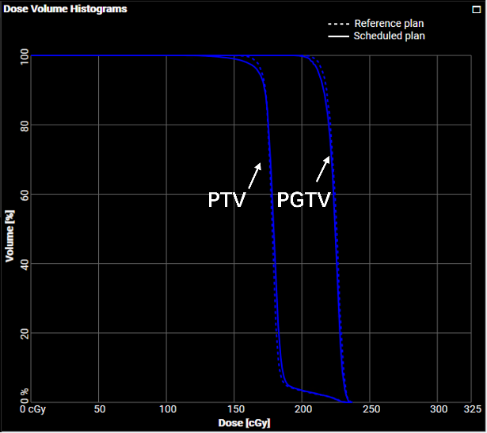 | 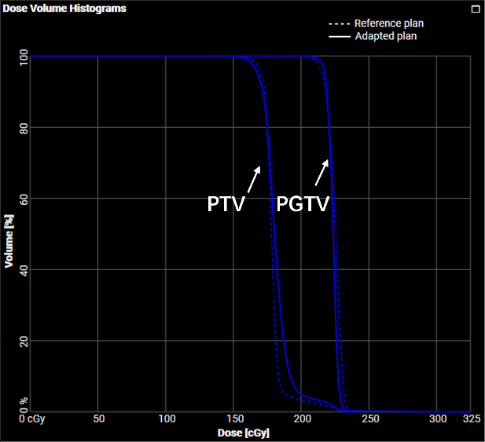 |
| --- | --- |
| (A) | (B) |
| 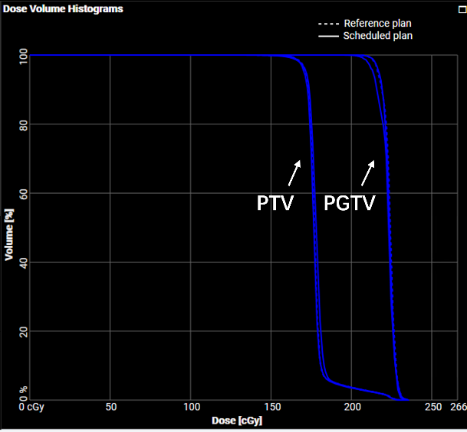 | 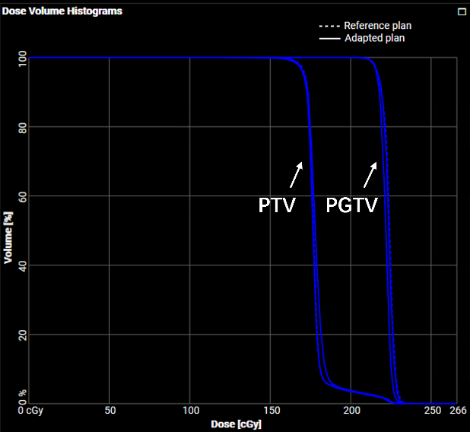 |
| (C) | (D) |

Supplementary Figure 2. Dose volume histogram (DVH) comparison of PTV and PGTV. (A) Scheduled plan, second session; (B) Adaptive plan, second session; (C) Scheduled plan, fourth session; (D) Adaptive plan, fourth session.
